# 

**Published:** 1863-12

**Authors:** 


### BOOKS RECEIVED.

*Transactions of the Medical Society of the State of New York, for the year* 1863, *from* J. D. Willard, *Secretary.*

*Transactions of the Ohio State Medical Society,* 1863, Edward B. Stevens, *Secretary.*

*Regimental Surgeons of the State of New York in the War of the Rebellion* 1861-3, *By* Sylvester D. Willard, M. D., *of Albany.*

